# Metacognitive Therapy for Alcohol Use Disorder: A Systematic Case Series

**DOI:** 10.3389/fpsyg.2018.02619

**Published:** 2018-12-19

**Authors:** Gabriele Caselli, Francesca Martino, Marcantonio M. Spada, Adrian Wells

**Affiliations:** ^1^Studi Cognitivi, Cognitive Psychotherapy School, Milan, Italy; ^2^School of Applied Sciences, London South Bank University, London, United Kingdom; ^3^Sigmund Freud University Milano, Milan, Italy; ^4^Department of Medical and Surgical Sciences DIMEC, University of Bologna, Bologna, Italy; ^5^Division of Clinical and Health Psychology, University of Manchester, Manchester, United Kingdom; ^6^Greater Manchester Mental Health NHS Foundation Trust, Manchester, United Kingdom

**Keywords:** addiction, alcohol use disorder, metacognition, metacognitive therapy, outcome, treatment

## Abstract

Alcohol Use Disorder (AUD) is a debilitating condition with serious adverse effects on health and psycho-social functioning. The most effective psychological treatments for AUD show moderate efficacy and return to dysregulated alcohol use after treatment is still common. The aim of the present study was to evaluate Metacognitive Therapy (MCT) as applied to AUD. Five patients were treated using a non-concurrent multiple baseline design with follow-up at 3- and 6-months time points. Each patient received 12 one-hour sessions of MCT. Following MCT all patients demonstrated large and clinically meaningful reductions in weekly alcohol use and number of binge drinking episodes that were upheld at follow-up in almost all cases. Metacognitive beliefs, as secondary outcome, also changed substantially. The findings from this study offer preliminary evidence of positive effects associated with MCT in AUD and support the need for a definitive trial of MCT in addictive behaviors.

## Introduction

Alcohol Use Disorder (AUD) involves loss of control over alcohol use, a strong desire or urge to use alcohol, and continued alcohol use in hazardous situations despite awareness about of persistent or recurrent life problems caused by the effects of alcohol (DSM-5, American Psychiatric Association [APA], 2013). The harmful use of alcohol is one of the world’s leading health risks and has been implicated in 5.9% of deaths globally (World Health Organization, 2014). Harmful alcohol use has also been associated with a wide range of mental health and social problems such as suicide (Merrill et al., 1992; Demirbas et al., 2003), increased risk of major depression (Boden and Fergusson, 2011), domestic violence (Leonard, 2001), child abuse (Widom and Hiller-Sturmhofel, 2001), and workplace absenteeism (Bacharach et al., 2010). A wide range of approaches have been developed to conceptualize and treat this disorder. Among them cognitive and behavioral models have highlighted the role of learning processes, cognitive biases and dysfunctional beliefs in the etiology and maintenance of AUD. One of the core principles underlying cognitive-behavioral therapy (CBT) for AUD is that alcohol serves as a powerful reinforcer of behavior. Over time, positive (e.g., enhancing social experiences) and negative (e.g., reducing negative affect) reinforcing effects of using alcohol become associated with a variety of internal and external stimuli. The cognitive component of these approaches highlights the role of barriers to change, such as biases, beliefs and expectancies which maintain alcohol use as a coping strategy to deal with negative affect or to reach desired goals. CBT aims to reduce the strongly reinforcing effects of alcohol by: (1) identifying the problematic situations that lead to alcohol use and teaching patients coping-skills to manage them (e.g., assertion drink refusal skills training); (2) increasing engagement in activities that are not related to alcohol use; and (3) removing motivational and cognitive barriers to change (e.g., Kadden, 2001; Donohue et al., 2004; Baillie et al., 2013). On the basis of these principles, a series of CBT protocols for AUD were developed and were extensively evaluated (Marlatt and Gordon, 1985; Monti et al., 1993; Kadden, 1995, 2001) with both abstinence and controlled drinking as treatment goals (e.g., Sanchez-Craig et al., 1984).

The CBT approach has provided valuable insights in the conceptualization and treatment of AUD, however, it is not without limitations. A central limitation of the behavioral component of CBT is that it does not elucidate why only a small proportion of individuals who use alcohol end up losing control over their use. A central limitation of the cognitive component of CBT is the failure to establish if irrational beliefs play a causal role in the etiology and development of AUD rather than being an epiphenomenon of this condition. These structural weaknesses of CBT as applied to AUD may explain its moderate effectiveness when compared to other forms of treatment, including medical management, treatment as usual, or active psychosocial treatments (e.g., Project Match Research Group, 1997; Burtscheidt et al., 2002; Balldin et al., 2003; Litt et al., 2003; Wetzel et al., 2004; Anton et al., 2005; Wolwer et al., 2011; Farren et al., 2014). In addition, treatment effects for CBT appear to diminish over time, especially at 6- to 9-month follow-up (Magill and Ray, 2009).

Drawing on the S-REF model (Wells and Matthews, 1994) it has been argued that a possible reason for CBT’s lack of efficacy might be due to residual symptoms and mechanisms that remain present at a metacognitive level (Spada and Wells, 2008; Spada et al., 2009, 2015). Specifically, the modification of the content of biased cognitive beliefs, which is the main focus of CBT, does not directly modify metacognitive beliefs presumed to be driver of maladaptive cognitive processes (e.g., worry, rumination, desire thinking) as implicated in the S-REF.

Over the last twenty-five years the Self-Regulatory Executive Function (S-REF) model has offered novel insights into the role of metacognition in psychopathology (Wells and Matthews, 1994, 1996; Wells, 2000). Central to the S-REF model are the processes which monitor, generate and maintain intrusive and biased cognitive experiences (Wells, 2009). The S-REF model has led to a novel form of psychological therapy, Metacognitive Therapy (MCT; Wells, 2009), which has been applied to the treatment of anxiety and depression with notable results (e.g., Normann et al., 2014). From the metacognitive standpoint, psychological disturbances are maintained by the activation of the Cognitive-Attentional Syndrome (CAS) under conditions of distress. The CAS encompasses repetitive negative thinking styles (rumination and worry), thought suppression, maladaptive threat or self monitoring, and avoidance. The activation of the CAS brings an increase of attentional focus toward distress congruent information and feedback loop which fail to regulate threatening thoughts. The activation, perseveration and escalation of the CAS is linked to the presence of unhelpful metacognitive beliefs. These are beliefs about thinking and ways in which thinking can be controlled. Metacognitive beliefs are either positive (e.g., “Worrying will help me cope”) or negative (e.g., “Thoughts are dangerous and should be controlled”) and are associated to generic plans for guiding cognition and behavior.

Research undertaken over the last decade has proposed AUD may be conceptualized using this metacognitive perspective (Spada and Wells, 2005, 2006; Spada et al., 2013, 2015; Caselli et al., 2013b). According to this view, it has been argued that metacognitive beliefs lead to the activation of CAS components associated with AUD (such as perseverative thinking about alcohol-related intrusions, the monitoring of internal or external alcohol-related cues, and the reduction of adaptive metacognitive monitoring). Emerging evidence has supported this conceptualization when applied to different forms of perseverative thinking (e.g., desire thinking, rumination and worry) shown to be highly associated with craving and levels of alcohol use in both non-clinical and clinical samples through cross-sectional designs (Caselli et al., 2008, 2012; Goldsmith et al., 2009; Smith and Book, 2010; Caselli and Spada, 2011, 2015; Chakroun-Baggioni et al., 2017), experimental studies (Caselli et al., 2013a,b, 2017; Caselli and Spada, 2011, 2015, 2016) and longitudinal research (Caselli et al., 2010; Martino et al., 2017).

The detrimental interplay between alcohol use and adaptive metacognitive monitoring, another element of the CAS, is widely accepted. In particular impairment of attentional functioning appears to play a fundamental role in determining alcohol effects. For example, alcohol’s pharmacological properties can narrow the perception to immediate cues and reduce the capacity for abstract reasoning (Steele and Josephs, 1990). In addition, alcohol reduces self-awareness, conceptualized as the ability to attribute self-relevance in encoding information (Hull, 1981) and neuroscientific evidence suggests that alcohol intoxication impairs neurological systems associated to meta-level processing (Nelson et al., 1998). All these processes are likely to play a relevant role in the effective monitoring of internal states once a drinking episode has started (Spada and Wells, 2006; Spada et al., 2007b). An ineffective monitoring of internal states (termed “metacognitive monitoring”; Spada and Wells, 2006) can lead to higher levels of alcohol use because information on emotional change (e.g., feeling relaxed) and proximity to goals of alcohol use (e.g., achieving a greater level of relaxation) that would serve as a stop signal is not attended to.

The links between metacognitive beliefs and aspects of the CAS in AUD is also now extensive. Research linking metacognitive beliefs on the one hand, and forms of perseverative thinking on the other, is well-established (e.g., Cartwright-Hatton and Wells, 1997; Wells and Papageorgiou, 1998; Papageorgiou and Wells, 2003; Wells and Cartwright-Hatton, 2004). This association has been extensively explored in AUD with similar findings (Spada and Wells, 2005; Spada et al., 2007b; Caselli and Spada, 2010, 2011). For example, cross-sectional research using self-report instruments has demonstrated that metacognitive beliefs are elevated in problem drinking (Spada and Wells, 2009). Furthermore, a longitudinal study showed how beliefs about the need to control thoughts predict levels of alcohol use and relapse at 3, 6, and 12 months in a sample of problem drinkers (Spada et al., 2009). Finally, in research aimed at uncovering the structure of alcohol-specific metacognitive beliefs in problem drinkers, both positive and negative metacognitive belief systems related to alcohol use were identified (Spada et al., 2007a; Spada and Wells, 2008, 2009).

Taken together, these data support the applicability of the S-REF model to understanding the development and maintenance of AUD and suggest that metacognitive therapy (MCT, Wells, 2009) may be beneficial in treating it. A recent study examined whether a specific MCT technique, detached mindfulness, would be more effective than a control condition in reducing negative meta-appraisal of alcohol-related thoughts, the conviction in maladaptive metacognitive beliefs, and associated distress level and urge to use alcohol in a small sample of patients with AUD (Caselli et al., 2016). Findings suggested that detached mindfulness was associated with a faster change in status. This implies that a targeted focus on modifying the relationship to one’s thoughts (rather than simply habituating to them) may be of benefit. The findings also support a broader and more extensive application of a whole MCT package for patients with AUD.

The present study aimed at examining the effects associated with a brief course of MCT in a series of patients with AUD. The main goal of the treatment was controlled or reduced-risk alcohol use. This was suggested as a pragmatic option, with a view to sustain patient engagement, because abstinence as a goal can often represent a barrier (Connor et al., 2016). In addition, from a metacognitive perspective, directly sustaining a controlled drinking goal is more likely to enhance metacognitive control which would otherwise not be achieved through abstinence because negative metacognitive beliefs about the uncontrollability of behavior and thoughts need to be tested through controlled behavior (i.e., continued and controlled alcohol use).

## Methods

### Design

This case series adopted a non-concurrent multiple baseline (MB, Watson and Workman, 1981) design across individuals with follow-up in order to: (1) test the feasibility and replicability of MCT across different individuals with AUD; and (2) examine if MCT is associated with positive outcomes in these cases. The MB is a well-established design with a wide range of applications and a multitude of publications in the clinical field supporting its use and validity. MB is commonly used in cases where the dependent variable is not expected to return to normal after treatment has been applied (Carr, 2005). This kind of design can offer important advantages. Firstly, repeated measures can help to establish the prediction of a baseline’s data path into the subsequent treatment phase and allow for the detection of a difference between the actual data path in treatment and the path predicted from baseline. Secondly, this effect can be replicated across different participants independently of the baselines’ length. A detailed data collection with several time points and different baseline length can control for maturation, exposure to the clinical setting, repeated testing, and regression to the mean, increasing the confidence that any observable changes are attributable to the intervention. A predetermined set of baseline lengths was randomly selected and assigned to the five patients of the present study. Baseline length ranges were 3–7 weeks. Treatment was initiated at the predetermined time only if baseline was stable, otherwise extension of the baseline was planned. Stability was defined as an absence of a decreasing trend of at least two consecutive data-points prior to introduction of treatment. Treatment was constituted by 12 one-hour-long sessions as this timeframe had been found to be sufficient to complete the MCT protocol in pilot work. Following the screening assessment, patients received questionnaires on a weekly basis with a view to monitor alcohol use, number of binge drinking episodes and symptoms levels. Following the baseline period, MCT was delivered on a weekly basis. After treatment, patients were followed up at 3 and 6 months, no additional treatment was delivered during the follow-up period. The goal of MCT was to control alcohol use.

### Participants

Patients included in this study were the first five consecutively assessed individuals who met the following criteria: (1) primary diagnosis of AUD as determined by the SCID-5 (First et al., 2015); (2) age 18–65 years; (3) absence of borderline personality disorder; (4) absence of a concurrent psychological treatment; (5) no evidence of physical withdrawal syndrome; (6) no evidence of progressive cerebral traumas or severe cognitive deficits; (7) not actively suicidal; (8) medication free; (9) no concurrent substance use (apart from nicotine) in the previous 6 months; and (10) clear understanding of the Italian spoken language. These criteria were evaluated by the second author and a trained psychologist independently.

#### Patient 1

Patient 1 was 25 to 30-years old and reported difficulties in regulating drinking especially during his job. He reported problematic alcohol use since his early teenage years, often associated with cannabis and/or cocaine use. He reported that alcohol use had been the main problematic issue in his life and gave him much trouble (e.g., law problems, brawls, problematic issues in both intimate relationships and workplace). He tried to reduce and/or stop alcohol use many times without long lasting positive outcomes. He reported contacting mental health services, both public and private, often driven by his family. He always rejected pharmacological treatment. He previously undertook 6 months of psychotherapy without any results and he was unable to define the approach employed. The patient also got in contact with Alcoholics Anonymous but abandoned after 2–3 meetings. He met criteria for Major Depressive Disorder of moderate severity.

#### Patient 2

Patient 2 was 25 to 30-years old and reported difficulties in controlling alcohol use that began almost 10 years previously as a means of managing anxiety in social situations. He reported that he began to consider his alcohol use as problematic 2 years earlier, when he had some relational problems with his girlfriend and friends because of his behavior when drunk. He attempted, over the last 2 years, to reduce alcohol use autonomously with some transient positive results but he experienced recurrent relapses. At the beginning of pre-treatment, he thought he had completely lost control over his alcohol use. In addition, he met the criteria for Social Anxiety Disorder. He was medication free and he had never had contact with mental health services.

#### Patient 3

Patient 3 was 35 to 40-years old, he was unemployed and reported he was not able to continue his job because of drinking problems. He also reported that difficulties surrounding alcohol use started to become serious 12 years prior, with binge drinking episodes pre-dating this time. During last 10 years he began using alcohol when alone, and on a daily basis, and this habit gradually led to a reduction of social contacts and general withdrawal. He also met criteria for Major Depressive Disorder.

#### Patient 4

Patient 4 was 60 to 65-years old and reported that stress related to his job and family difficulties were the main reason for his alcohol misuse. He reported he had never consumed too much alcohol until 10–15 years ago. He was unable to define a specific change in his life circumstance associated with his change in alcohol use, but he reported a general increase in life and work problems occurring at the time. At present, his excessive alcohol use persisted no matter how he tried to reduce it. He got in contact with mental health services during the previous 2 years, but he rejected both the goal of abstinence and pharmacological therapy with Disulfiram. He attended a handful of psychotherapy sessions but did not feel these were effective and he dropped out.

#### Patient 5

Patient 5 was 35 to 40-years old and reported that recent problems with alcohol use had lasted 3 years and that drinking too heavily had featured intermittently since teenage years. The patient met criteria for dysthymia but was medication free. The only previous contact with mental health services was 2–3 assessment sessions with a psychotherapist 5 years ago. Patient reported having used other substances (cocaine) but not in the last 12 years.

### Outcome Measures

#### Alcohol Use Disorders Identification Test Consumption (AUDIT-C; Bush et al., 1998)

The AUDIT-C includes items 1 to 3 of the 10-item AUDIT which assess alcohol use. Individuals select one of five statements (per question) that most applies to their alcohol use. Responses are scored from 0 to 4 with higher scores representing higher levels of problematic alcohol use. The summary score for the total AUDIT-C ranges from 0, indicating no presence of problematic alcohol use, to 12 indicating severe levels of problematic alcohol use. This self-report measure has been extensively adopted and possesses a well-established validity and reliability (Bush et al., 1998). The Italian version of the measure was used in the current study (Piccinelli et al., 1997).

#### Hospital Anxiety and Depression Scale (HADS; Zigmond and Snaith, 1983)

The HADS consists of 14 items on a 4-level Likert scale that refer to how respondents have been feeling over the past week (from “Most of the time” to “Not at all”). The HADS includes two sub-scales (seven item each) assessing anxiety and depression. Higher scores represent higher levels of anxiety and depression. Overall, the scale possesses good validity and reliability and has been widely adopted in a wide range of clinical and non-clinical research settings (Zigmond and Snaith, 1983; Herrmann, 1997; Mykletun et al., 2001; Alati et al., 2004; Wagena et al., 2005). The Italian version of the measure was used (Costantini et al., 1999) which shows a good reliability with alpha equal to 0.89 and 0.88 for anxiety and depression sub-scales, respectively.

#### Positive Alcohol Metacognitions Scale (PAMS; Spada and Wells, 2008)

The PAMS consists of 12 items which assess positive beliefs about the need to use alcohol as a cognitive and emotional self-regulation strategy (metacognitive beliefs). Higher scores indicate higher levels of positive metacognitive beliefs. The PAMS possesses a reliable factor structure and good internal consistency and validity in both clinical and non-clinical samples (Spada and Wells, 2008). The Italian version of the measure was used (alpha = 0.88; Di Blasi et al., 2013).

#### Negative Alcohol Metacognitions Scale (NAMS; Spada and Wells, 2008)

The NAMS consists of six items which assess negative metacognitive beliefs about uncontrollability and cognitive harm of alcohol use. Higher scores indicate higher levels of negative metacognitive beliefs about alcohol use. The NAMS possesses a reliable factor structure and good internal consistency and validity in both clinical and non-clinical samples (Spada and Wells, 2008). The Italian version of the measure was used (alpha = 0.75; Di Blasi et al., 2013).

#### The Penn Alcohol Craving Scale (PACS; Flannery et al., 1999)

The PACS is a 5-item scale that assesses the level of craving for alcohol. Its items refer to duration, frequency, intensity and uncontrollability of craving plus an overall evaluation of the subjective experience of craving during the previous week. Each question is scaled from 0 to 6. This instrument has been shown to possess good psychometric properties (Flannery et al., 1999). The Italian version of the measure was adopted (alpha = 0.80; Caselli and Spada, 2011).

#### Quantity Frequency Scale (QFS; Cahalan et al., 1969)

This QFS consists of nine items assessing levels of alcohol use, with three sub-scales assessing the use of beer, spirits and wine. The total scores from the different sub-scales are added to estimate weekly level of alcohol use. The QFS has been extensively used and possesses good reliability and validity (Hester and Miller, 1995). This instrument was completed on a weekly basis, referring to the previous week’s alcohol use.

#### Cognitive Attentional Scale – Alcohol (CAS-A)

A self rating scale was constructed for this study to assess dimensions of the CAS and related metacognitive beliefs that are usually associated with AUD. Items that referred to CAS components included: (1) time spent ruminating on alcohol-related thoughts; (2) associated distress; and (3) number of binge drinking episodes. Questions on metacognitive beliefs included 10 items referring to both positive metacognitive beliefs (e.g., “I need to drink in order to control my thoughts”) and negative metacognitive beliefs (e.g., “I have no control over my drinking”). All dimensions apart from number of binge drinking episodes were rated for the past week on 0–100 scales. The psychometric properties of this instrument have not been evaluated.

### Procedure

We sought and obtained ethics approval for the study from the Ethics Committees of Studi Cognitivi Research Institute Ethics and the School of Applied Sciences at London South Bank University (UREC1503). Participants referred for alcohol-related problems to outpatient clinics in Milan and Modena were invited for an assessment interview in order to determine eligibility for the study. The same invitation was offered to those who had directly contacted the project lead after seeing leaflets and web announcements. All patients were assessed independently by the second author and a psychologist to confirm the diagnosis of AUD and evaluate inclusion and exclusion criteria. After agreement between assessors and informed consent were obtained, an initial and complete assessment was administered. Four participants were excluded from the study because of presence of Borderline Personality Disorder (2 participants) and lack of a primary diagnosis of AUD (2 participants). Weekly ratings were taken for the QSF and CAS-A over the baseline period. The self-report questionnaires were administered to patients on a weekly basis. Once the predetermined baseline length was reached, a fuller assessment was conducted which involved the administration of all self-report measures to be repeated at post-treatment and at 3 and 6-months follow-up. During treatment, QFS and CAS-A were completed at the beginning of each session.

### Treatment

The MCT protocol for AUD consisted of 12 weekly sessions of 45–60 min duration and followed the core MCT steps as developed by the fourth author (Wells, 2009) adapted to the metacognitive formulation of AUD (Spada et al., 2013, see Table 1). In the first treatment session an idiosyncratic case formulation based on the metacognitive model of AUD was presented as a basis for a socialization to the model that followed. The latter emphasized how dysregulation of drinking behavior can be caused by alterations in self-monitoring and negative metacognitive beliefs about uncontrollability. Socialization was strengthened by the use of Socratic dialog (e.g., “If you discovered that you had control over your alcohol use how much of a problem would remain?”) and the use of metaphors. At the end of the first treatment session Adaptive Self-Monitoring (ASM) was introduced as a method to discover the degree of control patients may have over their alcohol use. ASM is an attentional refocusing strategy that involves the orientation of attentional focus toward goal-progress information as it can give appropriate feedback to the cognitive system on when goals are reached, and ongoing drinking behavior can be moderated or stopped. This type of ASM is present in everyday life. For example, the monitoring of an appropriate highway exit to reduce our vehicle speed, change our route, and reach our destination, or the monitoring of cooking time and food appearance to define when to stop cooking. In the case of AUD, it implies focusing on global self and desired goals during alcohol use or simply counting the number of empty glasses on the table. ASM exercises were practiced in session to deliver appropriate information and feedback on self-regulation. Patients were then asked to freely practice ASM as homework.

**Table 1:** Summary table of the MCT protocol for AUD.

Sessions	Contents
1	Case formulation Socialization
2–4	Challenge metacognitive beliefs about the uncontrollability of alcohol use Strengthen adaptive self-monitoring Metacognitively delivered drinking control experiments Verbal reattribution strategies
5–6	Challenge metacognitive beliefs about the uncontrollability and dangerousness of thoughts Detached mindfulness techniques Metacognitively delivered exposure and response postponement Verbal reattribution strategies
7–8	Challenge “alcoholic brain” beliefs Evidence examination and mini-survey Verbal reattribution strategies
9–10	Challenge positive metacognitive beliefs about alcohol use Verbal reattribution Behavioral experiments
11–12	Relapse prevention

In the following seven sessions, treatment focused on careful identification of which negative metacognitive beliefs about uncontrollability and/or danger were present and on modifying them. Negative metacognitive beliefs about uncontrollability and danger showed different facets: (1) uncontrollability of alcohol use (“I cannot stop using alcohol when I start”); (2) uncontrollability of thinking about alcohol use (“I cannot stop thinking about using alcohol”); (3) thought-action fusion (“Thoughts about alcohol will make me drink”); (4) abnormal brain beliefs (“I have no control over alcohol use because my brain is abnormal in some way”). Each of these metacognitive beliefs became the target of MCT interventions when present. The application of ASM, controlled drinking experiments, and verbal reattribution were adopted to modify beliefs about uncontrollability of alcohol use. The application of detached mindfulness techniques (Wells, 2009), postponement of perseverative thinking such as rumination, and verbal reattribution were used to modify beliefs about uncontrollability of thinking about alcohol use. Detached mindfulness, metacognitive delivered exposure to thoughts relating to alcohol use with response postponement and verbal reattribution were used to modify beliefs about thought-action fusion. Verbal reattribution, especially the examination of evidence and counterevidence, and mini-surveys were used to modify abnormal brain beliefs.

In the next two sessions positive metacognitive beliefs about alcohol use became the focus of treatment. To counteract these beliefs an analysis of evidence and counterevidence was undertaken to reinforce knowledge about how the desired outcomes could be better achieved in other ways and behavioral experiments were applied to test this.

In the last two treatment sessions the intervention focused on relapse prevention and the further reappraisal of metacognitive beliefs. This included metacognitive beliefs about the meaning of lapses and relapses. Relapse prevention involved the construction of a replacement plan for situations where using alcohol may take place.

### Training

All patients were treated by the first author who is a Level-2 registered MCT therapist and received training and ongoing supervision in MCT from Professor Adrian Wells.

### Data Analysis and Clinical Significance

**FIGURE 1:**
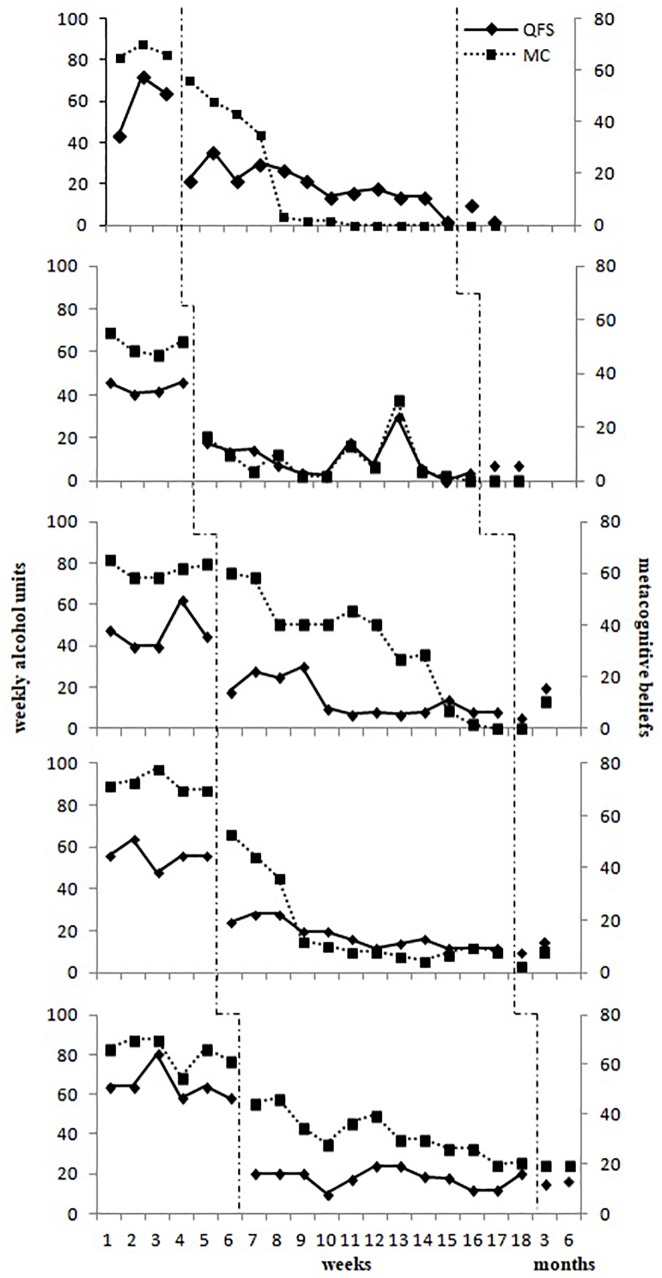
Scores on the QFS and Metacognitive Beliefs (MC) as measured by the CAS-A across baseline, metacognitive therapy and follow-up for each patient.

The primary goal of this case series was to determine if there is a clear treatment effect following the introduction of MCT. Typically, the visual examination of graphed data provides a reliable test of the treatment effect because only unambiguous effects are likely to be present (Parsonson and Baer, 1992). Weekly scores across baseline, treatment and follow-up on the QFS and metacognitive beliefs are presented in Figure 1. In addition, pre-treatment, post-treatment and follow-up scores on standardized measures for each of the five patients are presented in Table 2.

**Table 2:** Descriptive statistics (mean and standard deviation) on the main outcome measures across the five cases at pre-treatment, post-treatment and follow-up.

	Pre-treatment	Post-treatment	3-months follow-up	6-months follow-up
QFS	53.8 (8.14)	9.2 (7.16)	9.6 (3.6)	12 (7.07)
AUDIT-C	8.4 (1.14)	3.2 (0.45)	3.0 (0.71)	3.0 (0.71)
HADS-Anxiety	13.0 (4.00)	6.0 (3.39)	6.6 (4.28)	6.0 (2.35)
HADS-Depression	12.6 (3.85)	6.6 (2.88)	6.4 (3.51)	3.8 (1.64)
PACS	15.4 (3.97)	5.4 (3.85)	3.4 (3.21)	4.2 (4.44)
PAMS	24.6 (5.98)	16.0 (1.12)	14.4 (2.51)	15.4 (2.30)
NAMS	17.6 (1.14)	8.4 (1.52)	9.2 (2.17)	8.8 (1.30)

*QFS, quantity frequency scale; AUDIT-C, alcohol use disorders identification test consumption; HADS, hospital anxiety and depression scale; PACS, penn alcohol craving scale; PAMS, positive alcohol metacognitions scale; NAMS, negative alcohol metacognitions scale*.

To determine whether a change over the course of treatment was clinically significant we adopted a two-fold criterion (Jacobson and Truax, 1991; Bauer et al., 2004). Following this method each patient was allocated to one of four outcomes: reliable deterioration, no change, reliable improvement or recovered. The first three outcomes are derived from the combination of different statistical approaches to reliable change. The Reliable Change Index (RCI, Bauer et al., 2004) approach, which determines whether the change is statistically significant, was applied to AUDIT-C scores. Data to calculate the RCI for the AUDIT-C score was drawn from a large sample of the general population (Aalto et al., 2009), and a minimum change of 3.46 points on AUDIT-C was consequently defined as a reliable change.

To be classified as recovered, patients would have had to demonstrate reliable change on their post-treatment or follow-up scores with these being below a clinical cut-off point for each of the primary outcome measures: (1) AUDIT-C; (2) QFS; and (3) number of DSM-5 criteria for AUD. With reference to the AUDIT-C score, different cut-offs have been established in different countries on the basis of sensitivity and specificity (Anderson et al., 2005): in Italy, total scores equal to or greater than five for men and four for women indicate possible hazardous consumption of alcohol(Struzzo et al., 2006). Data to establish a clinical cut-off for QFS was drawn from the recommendation of the Italian Ministry of Health that defines a safe weekly alcohol use of under 14 weekly units for men and 7 weekly units for women (Società Italiana di Nutrizione Umana [Sinu], 2014). Finally, for DSM-5 criteria for AUD none should have been met for at least 3 months but for less than 12 months (with the exception of craving). This was defined as an established threshold for early remission in line with the specifier for individuals previously diagnosed with AUD (American Psychiatric Association [APA], 2013).

## Results

### Primary Outcomes

Weekly alcohol use appeared relatively stable during the baseline period for all patients (see Figure 1). Scores remained constantly above the limit of two standard deviations over the normative mean for the Italian population (Kehoe et al., 2012) as indicated by a normative comparison approach (Jacobson et al., 1986). The weekly mean number of binge drinking episodes at baseline was 3.3 (*SD* = 1.5) for patient 1, 1.5 (*SD* = 0.6) for patient 2, 2.4 (*SD* = 0.5) for patient 3, 3.4 (*SD* = 0.5) for patient 4, and 4.5 (*SD* = 0.8) for patient 5. When the treatment was introduced weekly alcohol use and related metacognitive beliefs significantly reduced for all patients. During treatment there were few binge drinking episodes for all patients. In this group of patients, the main effect of treatment appeared in the first half of the treatment, which focused upon using strategies to acquire a greater degree of control over alcohol use. These were maintained in the second half of treatment, which was more focused on the consolidation of new metacognitive knowledge and on relapse prevention strategies. All participants maintained their gains at post-treatment and follow-up, with a level of weekly alcohol use relatively unchanged to that was established during treatment with the exception of Patient 3 who experienced an increase in weekly levels of alcohol use at 6-months follow-up but remined at a lower level compared with the baseline. The levels of weekly alcohol use at post-treatment and follow-up were within one standard deviation of the normative data for the Italian population for all patients. No binge drinking episodes were reported at post-treatment and at 3- and 6- months follow-up. The treatment was well-tolerated with no drop-outs and all patients reporting that it was helpful in gaining appropriate control over their alcohol use.

### Secondary Outcomes

**FIGURE 2:**
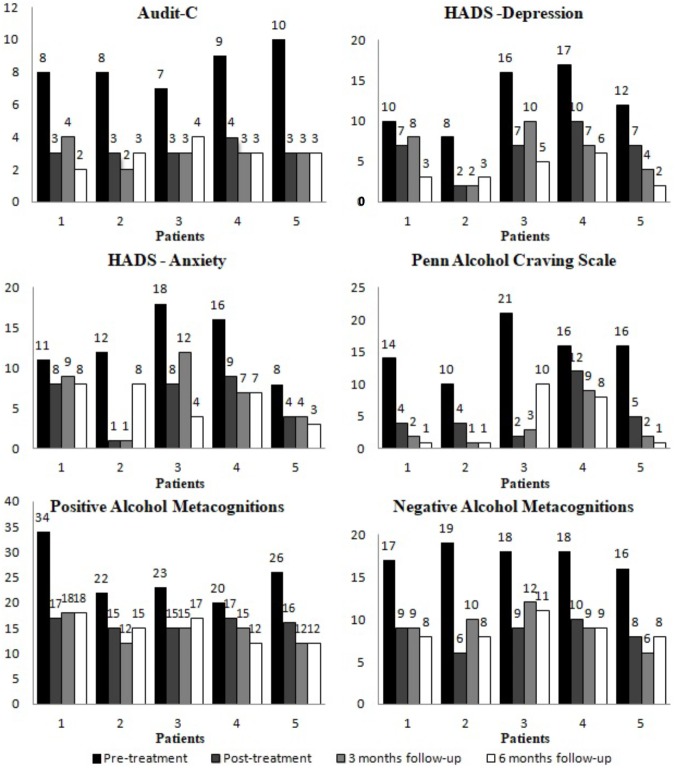
Scores on standardized measures at pre-treatment, post-treatment and follow-up for each patient.

The weekly measure of metacognitive beliefs did not change during baseline and showed a substantial reduction during treatment (see Figure 1). The decrease in the degree of conviction in metacognitive beliefs was quite rapid for Patients 2 and 5 after the beginning of the treatment. Patients 1 and 4 showed a more gradual decrease within the first half of treatment and remained stable in the second half. Patient 3 showed a constant decrease across treatment. These changes appeared stable at post-treatment and at 3- and 6- months follow-up. Scores for the PAMS and NAMS decreased at post-treatment and follow-up when compared to baseline scores and reached a level within one standard deviation of a non-clinical population as reported by Spada and Wells (2008) (See Figure 2). Scores on the PAMS and NAMS decreased, mirroring the weekly metacognitive beliefs measure changes. Similar results were replicated for anxiety, craving, and depression. Scores on HADS and PACS were lower at post-treatment and follow-up. Again, Patient 3 showed an increase in levels of craving at 6-month follow-up, but this remained lower compared to baseline.

### Clinical Significance

Each patient showed a reliable change for AUDIT-C with a change in score that ranged from 4 to 7 points. This reliable change was confirmed at 3- and 6- months follow-up for all patients with the exception of Patient 3 who reported an increase, but this remained stable when compared to pre-treatment scores. At post-treatment and follow-up all patients scored below the clinical cut-off for AUDIT-C and three patients reported weekly alcohol use below the QFS cut-off. Patient 5 showed a QFS over the safe cut-off at post-treatment (QFS = 20), 3-months follow-up (QFS = 5) and 6-months follow-up (QFS = 16). Patient 3 showed QFS below cut-off at post-treatment and at 3-months follow-up but an increase in weekly alcohol use (QFS = 20) at 6-months follow-up. However, levels of weekly alcohol use remained significantly lower for Patients 3 and 5 when compared to pre-treatment QFS scores. None of the DSM-5 criteria for AUD were met at post-treatment and follow-up by Patients 1, 2, 4, and 5, while Patient 3 met one criterion for AUD. Taken together these findings indicate that Patients 1, 2, and 4 were classified as recovered while Patients 3 and 5 were classified as improved.

## Discussion

The aim of this study was to evaluate the preliminary effects associated with MCT as a treatment for AUD. The outcomes of our study provide support the use of MCT as a therapeutic approach for AUD that may be associated with a clinically meaningful improvement in behavioral, cognitive, and affective self-regulation. Substantial reduction in weekly alcohol use and the absence of binge drinking episodes were observed for all patients compared to baseline. This change suggests an early remission from AUD for almost all patients. The reduction in symptoms appeared to remain stable in most cases at 3- and 6- follow-up.

Overall the treatment appears to have been successful and feasible with none of the patients reporting any worsening of psychological symptoms (anxiety and depression) and craving. Furthermore, our findings suggested that MCT might be a viable treatment for AUD as a primary diagnosis, at least with absence of physical withdrawal syndrome, especially when controlled drinking is an accepted or desired treatment goal.

Despite these encouraging results, several significant limitations of the current study need to be noted. Firstly, sample size was small, implying that the generalizability of measured effects should be considered with caution. Secondly, there was no control condition and so it is was not possible to partial out time effects and non-specific factors from the treatment effects. Thirdly, the delivery of treatment by a single individual means we cannot determining the impact of therapist factors on outcomes. Fourthly, the use of self-report measurements may have led to overestimation of treatment effects. Finally, the study lacked any formal assessment of adherence to treatment.

Overall, the outcome in this case series suggests that MCT is a feasible tretament with AUD and appears to be associated with reduced problematic drinking and increased control (at least in the short term). Future studies of MCT for AUD with larger samples and randomized designs are recommended in order to determine whether this approach is efficacious and whether it may provide an alternative to existing treatments.

## Ethics Statement

This study was carried out in accordance with the recommendations of LSBU code of practice for research with Human Participants, LSBU Research Ethics Committee (UREC 1503); with written informed consent from all subjects. All subjects gave written informed consent in accordance with the Declaration of Helsinki. The protocol was approved by the LSBU Research Ethics Committee (UREC 1503).

## Author Contributions

GC designed the research and treatment protocol, run therapy sessions, analyzed the data, and wrote the manuscript. FM designed the research protocol, assessed participants, and analyzed data. MS designed the research and treatment protocol, collected and analyzed data, and revised the manuscript. AW created theoretical foundation of the treatment, designed the research and treatment protocol, and revised the manuscript.

## Conflict of Interest Statement

The authors declare that the research was conducted in the absence of any commercial or financial relationships that could be construed as a potential conflict of interest.
